# Wear Behavior of Ceramic CAD/CAM Crowns and Natural Antagonists

**DOI:** 10.3390/ma10030244

**Published:** 2017-02-28

**Authors:** Ella A. Naumova, Stephan Schneider, Wolfgang H. Arnold, Andree Piwowarczyk

**Affiliations:** 1 Department of Biological and Material Sciences in Dentistry, Faculty of Health, Witten/Herdecke University, Alfred-Herrhausen-Strasse 44, 58455 Witten, Germany; wolfgang.arnold@uni-wh.de; 2 Department of Prosthodontic, Faculty of Health, Witten/Herdecke University, Alfred-Herrhausen-Strasse 44, 58455 Witten, Germany; Dr.Schneider.Stephan@gmx.de (S.S.); andree.piwowarczyk@uni-wh.de (A.P.)

**Keywords:** wear, CAD/CAM, full ceramic crowns, resin-matrix ceramics, glass-matrix ceramics, natural antagonist, volume loss, surface morphology

## Abstract

Objective: Evaluation of wear behavior of computer-aided design/computer-aided manufacturing (CAD/CAM) crowns from various restorative materials and natural antagonists. Method: Full CAD/CAM crowns fabricated with nanoceramic resin (Lava Ultimate (LU)), a glass ceramic in a resin interpenetrating matrix (Vita Enamic (VE)) and a lithium silicate reinforced ceramic enriched with zirconia (Vita Suprinity (VS)) were cemented on human molars. The crown and antagonists were subjected to simulated chewing. 3D data sets, before and after the chewing simulation, were generated and matched. Occlusal surface roughness, vertical and volume loss of the crowns and antagonists were analyzed. Results: Crown roughness was significantly different between the LU and VE groups after chewing simulation. Crown vertical loss differed in all groups. The highest crown volume loss was found in the LU group, and the lowest in the VE group. Comparisons between the LU and VE groups and the LU and VS groups were significantly different. The highest antagonist volume loss was reached in the VE group, the lowest was in the LU group. Conclusion: Roughness increased after chewing simulation. LU crowns are the most natural antagonist-friendly; these were the most susceptible to vertical and volume loss. Of the tested materials, the VE crowns are the most stable regarding occlusion.

## 1. Introduction

Engineering, physics, and material science have contributed to the technological developments for the progress of modern health care [1]. The two key technologies that opened new possibilities for restorative dentistry are the development of dental materials on the ceramic base and computer-aided design/computer-aided manufacturing (CAD/CAM) [2,3].

The aim of restorative dentistry is to find the appropriate biocompatible substitute for hydroxyapatite (HAP). Ceramic-glass-polymer materials are dental biomaterials with an interactive functionality. Full-contour CAD/CAM ceramic crowns are currently used and are a relatively new restoration method in the prosthodontic. There are different types of CAD/CAM block ceramic-glass-polymer materials [4] with different compositions and physical properties: IPS empress leucite ceramic, e.max press lithium disilicate ceramic [5], lithium disilicate glass-ceramic [6,7,8], Y-TZP ceramic [9], heat-pressed lithium disilicate ceramic, feldspathic porcelain, glass ceramic [2,10], zirconia, lithium disilicate, feldspathic ceramic [11], nanoceramic [4], resin nanoceramic [4,6], zirconia-reinforced lithium silicate ceramic (Vita Suprinity) [7], and polymer infiltrated ceramic network (PICN) materials, also called hybrid ceramics [4,12] or glass ceramic in a resin interpenetrating matrix (Vita Enamic) [13]. Gracis et al. (2015) created a classification system for dental ceramic materials that is useful for communication and educational purposes [13]. According to this system, all-ceramic materials and ceramic-like restorative dental materials are divided into three families with respect to the phase or phases present in their chemical composition as follows: (1) glass-matrix ceramics; (2) polycrystalline ceramics; and (3) resin-matrix ceramics [13].

It is well known that the most efficient function of two contact occlusal surfaces in the chewing system occurs when friction occurs between the occlusal surfaces consisting from HAP, which can be demineralized and remineralized, and as two tooth-antagonists are equally abraded. The restorative dental material must be appropriate for a wide spectrum of requirements: the materials must be cost-effective, easy to manufacture, durable, and biocompatible with the tooth build-up and the occlusal surface of the natural tooth-antagonist; they must also have excellent shade and light effects, good intraoral maintenance, hardness, and mechanical strength under pressure [2,14], as well as be flexible in strength and have a modulus of resiliency [15] of surface density with fracture, chemical, and wear resistance [2,14].

The occlusal surface roughness and smoothness of ceramic crowns are clinically important because these parameters have an impact on the plaque accumulation, wear kinetics, staining, the tactile perception of the patient [16], shade matching [12], and the wear of opposing natural teeth and opposing materials [8]. The wear of the ceramic crown occlusal surface can cause systemic effects if particles from the restorations are swallowed or inhaled [5]; the wear can also impact the thickness of the occlusal part of the full ceramic CAD/CAM crowns and the natural antagonists [2], which can lead to the occlusion change and an impaired aesthetic appearance and/or functional restrictions [5,17]. The decreased thickness and near-surface damage of the ceramic crowns limits their clinical success, especially on the posterior teeth [2].

A tribological study demonstrated that the roughness of the contacting surfaces influence their fatigue, friction, lubrication and wear [18,19]. There are many factors that influence the surface roughness and wear of restorative materials and the natural antagonists, such as the finishing [12] and polishing or glazing quality [8,9] of the occlusal surfaces of the CAD/CAM manufactured ceramic crowns, the flexural strength, modulus of resilience [15] and the solubility [20,21] of the ceramic materials, chewing with a different frequency, force of mastication, force profile, contact time, sliding movement, clearance of worn material [5], the hardness of food, pH of the saliva [22], gastroesophageal reflux disease [23], and toothbrushing [24].

In the literature, there is some data about the mechanical properties and wear resistance of the ceramic dental restorative materials with different compositions and physical properties. The examinations of the strength, solubility, surface and crystal structures of transformation-toughened zirconia (Y-TZP) [20], the fracture resistance of full-contour all-ceramic crowns [11] and ceramic disks [6], the fracture resistance of dental restoration ceramics using an edge chipping test [3], the flexural strength and modulus of resilience of the composite resin ceramic counterparts [15], the alteration of the surface roughness and gloss of all of the composite resin CAD/CAM blocks after toothbrushing [24], and the two- and three-body wear of the hybrid ceramic and feldspathic ceramic CAD/CAM blocks [25] have all been performed in vitro. However, there is no information about the wear properties or the three-dimensional wear assessment of the full CAD/CAM crowns made from new ceramic materials and their natural antagonist wear. The tribological concept of laboratory wear methods is important for the correlation to clinical wear [26].

Therefore, in the present in vitro study, chewing simulation, a contact wear method, was performed with a wet condition in artificial saliva. Because such a test-approach uses mechanical stress on the occlusal surface, it results in the wear of the occlusal part of the full ceramic crown and the natural antagonists. Therefore, the roughness, the vertical loss, and the volumetric loss were analyzed as wear parameters.

The null hypothesis for the present study was that different chemical types of glass-matrix and resin-matrix ceramics will not differ in their influence on the roughness and wear of the occlusal surface of the full ceramic crowns and the natural antagonists.

## 2. Results

### 2.1. Surface Roughness of the Full Ceramic CAD/CAM Crowns before and after Chewing Simulation

The comparison of the asfc values of the full ceramic CAD/CAM crowns occlusal surfaces roughness (*p* > 0.05) in the tested materials groups before and after the chewing simulation showed no statistically significant differences. All of the data are summarized in Table 1.

**Figure 1:**
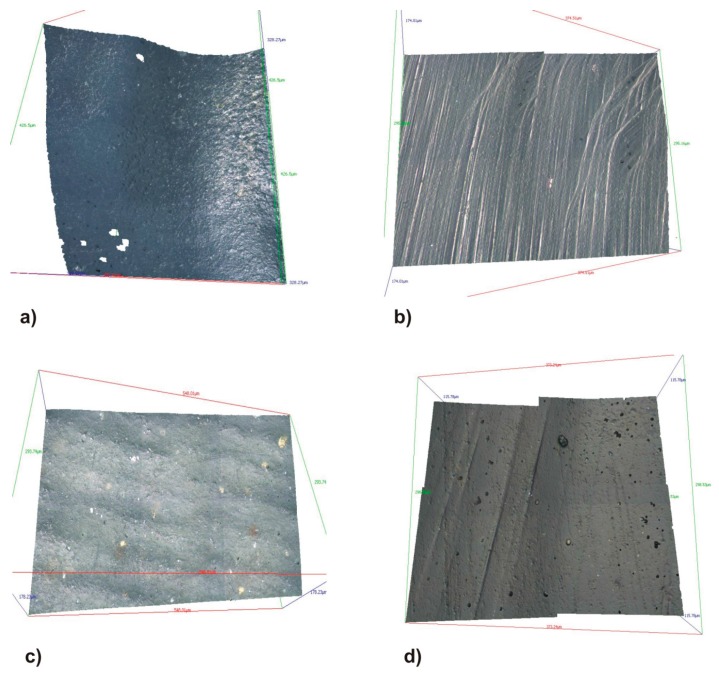

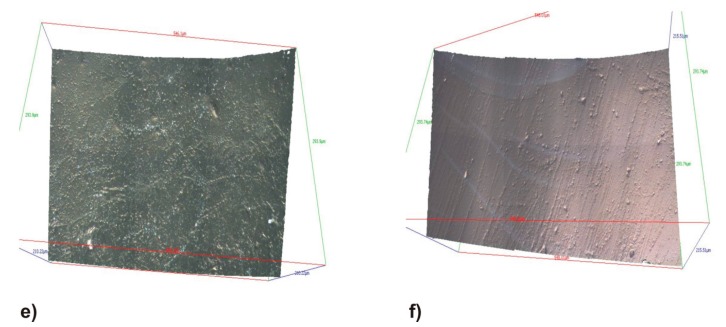
Three-dimensional surface images (stereolithographic (STL) files of the surface scan of the occlusal surfaces made from the different materials before and after chewing simulation: Lava Ultimate (**a**) before, (**b**) after; Vita Enamic (**c**) before, (**d**) after and Vita Suprinity (**e**) before, (**f**) after.

The comparison of the full ceramic CAD/CAM crowns occlusal surface roughness (*p* < 0.05) between the tested materials before the chewing simulation showed no statistically significant differences (Table 2, Figure 1); however, after the chewing simulation, statistically significant differences were observed between the Lava Ultimate and Vita Enamic groups (*p* = 0.043) (Table 2, Figure 1).

### 2.2. Vertical Loss of the Full Ceramic CAD/CAM Crowns and the Natural Antagonists 

**Figure 2:**
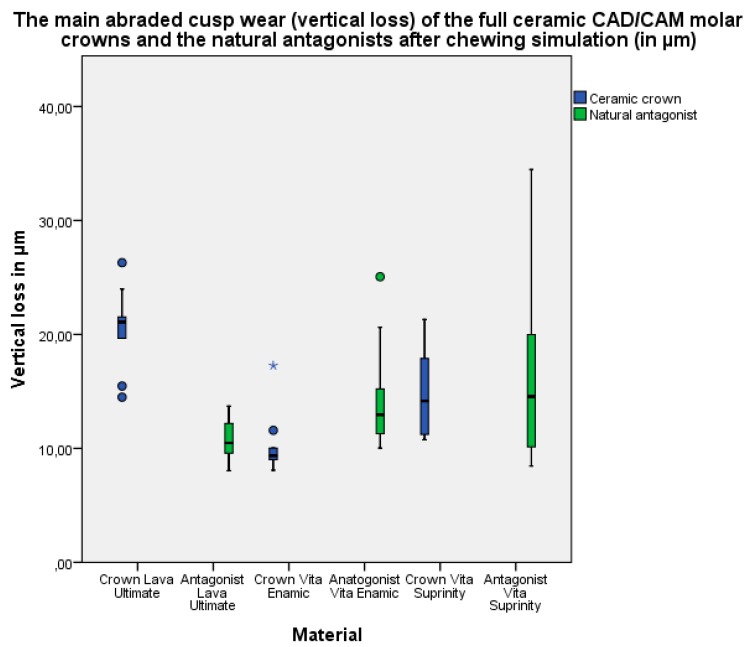
The main abraded cusp wear (vertical loss) of the full ceramic CAD/CAM molar crowns and the natural antagonists after chewing simulation (in µm).

The highest main abraded cusp wear (vertical loss) of the full ceramic CAD/CAM crowns after 1,200,000 chewing cycles was in the Lava Ultimate group 21.1 μm (interquartile range 18.6–24.5 μm), followed by the Vita Suprinity group 14.1 μm (11.1–18.4 μm), and the Vita Enamic group 9.3 μm (8.9–10.4 μm) (Figure 2).

A significant difference of the full ceramic CAD/CAM crowns vertical loss was found between all of the tested material groups (Table 3).

The vertical loss of the natural antagonists after 1,200,000 chewing cycles was found in the Vita Suprinity group 14.5 μm (interquartile range 9.7–20.8 μm), followed by the Vita Enamic group 12.9 μm (11.2–16.5 μm) and the Lava Ultimate group 10.4 μm (9.5–12.4 μm) (Figure 2). 

No significant differences in the median value of the vertical loss of the natural antagonists were found between all of the tested material groups; however, a tendency towards a difference between the Lava Ultimate and Vita Enamic groups was observed (Table 4).

### 2.3. Volume Loss of the Full Ceramic CAD/CAM Crowns and the Natural Antagonists 

**Figure 3:**
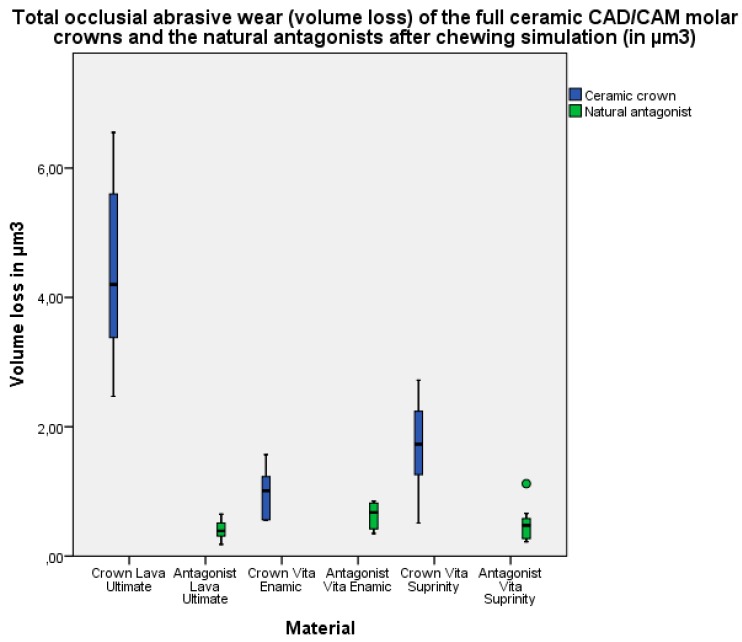
Total occlusial abrasive wear (volume loss) of the full ceramic CAD/CAM molar crowns and the natural antagonists after chewing simulation (in µm^3^).

The highest total occlusal abrasive wear (volume loss) of the full ceramic CAD/CAM crowns after 1,200,000 chewing cycles was in the Lava Ultimate group 4.2 µm^3^ (interquartile range 3.19–5.77 µm^3^), followed by the Vita Suprinity group 1.73 µm^3^ (1.14–2.25 µm^3^) and the Vita Enamic group 1.01 µm^3^ (0.55–1.25 µm^3^) (Figure 3).

A significant difference in the total occlusal abrasive wear (volume loss) of the full ceramic CAD/CAM crowns was found between the Lava Ultimate and Vita Enamic groups and between the Lava Ultimate and Vita Suprinity groups, but not between the Vita Suprinity and Vita Enamic groups (Table 5).

The highest total occlusal abrasive wear (volume loss) of the natural antagonists after 1,200,000 chewing cycles was in the Vita Enamic group 0.67 µm^3^ (interquartile range 0.42–0.82 µm^3^), followed by the Vita Suprinity group 0.47 µm^3^ (0.26–0.60 µm^3^) and the Lava Ultimate group 0.39 µm^3^ (0.29–0.53 µm^3^) (Figure 3).

A significant difference in the total occlusal abrasive wear (volume loss) of the natural antagonists was only found between the Lava Ultimate and Vita Enamic groups (Table 6).

## 3. Discussion

Progress in the material sciences substantially affects the dental sciences, especially in the progress of dental restoration [3]. The recent development of new and efficient dental ceramic restorative materials has made possible an improvement in the biocompatibility and wear characteristics of full ceramic CAD/CAM crowns that has not previously been achieved [2]. Clinical developments of dental restorative materials are affected by various clinical tasks during the dental restoration, including roughness and wear [2,14]. The limited availability of independent studies on the actual restorative dental materials makes it pertinent to evaluate their properties and to identify their potential strengths and limitations and their ability to achieve clinical success [15]. The consistency and correlation of in vitro and clinical studies are very important [5]. In most in vitro studies, flat polished ceramic specimens and prepared enamel from extracted molars as the antagonist were used [5]. The set-up of the in vitro study that consists of an unprepared enamel of molar cusps as a natural antagonist against glazed crowns is the most appropriate method to evaluate a ceramic material with regard to the antagonist wear [5]. The data of this study are important for the dental literature [26].

In the present study, measurements of the occlusal surface roughness of the full ceramic CAD/CAM crowns, before and after the chewing simulation, and a three-dimensional assessment of the wear of the crowns and the natural antagonists after the chewing simulation were made. Because wear is related to material properties such as hardness, flexural strength and load [19,27] in the present study physiological parameters of chewing pressure have been used. However, the results of the present study do not prove a simple correlation between material mechanical properties such as initial flexural strength, hardness, and wear. Other factors such as surface texture may play an important role.

To standardize the design of the present study, the CAD/CAM ceramic full crowns were fabricated in especially selected dental laboratories and were well-polished according the manufacture’s description. In the wear simulation concept, the method and devices should simulate processes that occur in the oral cavity during mastication [17]. Heintze et al. (2012) assumed that because the clinical wear process is a complex mechanism with different tribological phenomena occurring at the same time, there is no single laboratory wear method capable of producing a good correlation with clinical wear [26]. They proposed that if two or three methods that come very close to the clinical wear are combined, the correlation may be improved [26]. In the present in vitro study, the five-year chewing simulation used the contact mechanical Ivoclar wear method (Willytec chewing simulator, 1,200,000 cycles, 5 kg weight) [5] with a modification (300 mL artificial saliva with pH 7.0 in the test chamber) for the full ceramic CAD/CAM crowns and the natural antagonist was performed. The artificial saliva was used to simulate the oral cavity medium with a wet and natural solubility condition and the presence of worn material. In the previous studies, the test chamber was filled with water [5]. Following the FDA guidelines on good laboratory practice (GLP), the wear simulator used was a qualified machine; the force exerted by the hydraulic actuator during the dynamic loading of the flat specimens was controlled and regulated during all movements of the stylus, which is important for the qualitative experimental procedure [5]. To standardize the experimental procedure, chewing with the same contact time, frequency of mastication, force, force profile, and sliding movement was performed. 

In previous studies, the fracture resistance of the new restorative ceramic materials for the prosthodontic purpose was tested, but for ceramic disks [6] and for monolithic single crowns [11]. The wear resistance of the ceramic blocks made from Lava Ultimate and the Vita Enamic was analyzed by measuring the vertical substance loss [28]. We cannot compare these data directly with our results because the tests were not performed for CAD/CAM manufactured crowns. Chen et al. (2014) investigated the relationship between the restoration thickness to the fracture resistance of the Lava Ultimate and the IPS e.max CAD ceramic disks and determined that the tested materials with the thickness above 0.5 mm could withstand the normal bite force [6]. In the present study, the crown thickness was 1.5 mm. 

The materials paradigm or the processing-structure-properties relationships are important for understanding research areas such as biomaterials. Glass ceramic in a resin interpenetrating matrix (Vita Enamic) combines the advantageous properties of ceramic and composite materials. The theoretical predictions of glass ceramic in a resin interpenetrating matrix (hybrid ceramics) wear peculiarities [14] agree very well with the experimental results obtained in the chewing tests in the present study.

For glass ceramic in a resin interpenetrating matrix (Vita Enamic) and lithium silicate reinforced ceramic enriched with zirconia (Vita Suprinity), the loss of substance on the crowns and antagonists were found to be on a comparable level. For resin nanoceramic (Lava Ultimate), the wear of crowns was higher compared to the antagonists.

The limitations of the present study are as follows: the moving (chewing) pattern of the chewing simulator can only partially simulate the clinical situation; and the specimens were not submitted to terminal wear, to wear with hard particles, or to a toothbrushing device with a slurry of toothpaste or to chemical degradation.

Within the limitations of the present study, it might be suggested that glass ceramic in a resin interpenetrating matrix (Vita Enamic) is the most appropriate dental restorative material for full ceramic CAD/CAM crowns for molars.

The null hypothesis that different chemical types of glass-matrix and resin-matrix ceramics will not differ in their influence on the roughness and wear of the occlusal surface of full ceramic crowns CAD/CAM crowns for molars and the natural antagonists was not confirmed.

## 4. Materials and Methods

### 4.1. Tooth Collection, Preparation, and Storage

Tooth collection was approved by the ethical committee of Witten/Herdecke University (permission 116/2013). A total of 60 extracted, caries-free human molars with completed root growth (second dentition) were collected for this study (*n* = 30 maxillary molars and *n* = 30 mandibular molars). They were subdivided into three groups (*n* = 20) for each of the different ceramic materials. The mandibular molars (*n* = 10 in each group) were used for full ceramic crown preparation. The maxillary molars (*n* = 10 in each group) were used as the natural antagonists. After extraction, the teeth were cleaned of dental calculus and periodontal tissues using a scalpel, curette, and scaler. Subsequently, the teeth were gently polished with a rotating brush and a pumice and were stored in 0.9% sodium chloride (NaCl) containing 0.1% thymol at room temperature until use (a maximum of 3 months).

### 4.2. Abutment Preparation

The teeth in group 1 were prepared for full ceramic crowns. To avoid inter examiner differences, all of the tooth preparations were performed by the same operator, who was trained prior to the study by one of the authors. The teeth were fixed in a SE-580 15 plug (Combitech, Linköping, Sweden) with a bite registration material O-Bite (DMG, Hamburg, Germany). The circular chamfer, in compliance with a properly designed form and a convergence angle of approximately 6°, was prepared to achieve an ideal retention and resistance. Torpedo-shaped diamond burs CD72014 and CD72F012 (Busch, Engelskirchen, Germany) were used for the circular chamfer, and pear-shaped diamonds (831/014, 8831/014) (Busch, Engelskirchen, Germany) were used for the occlusal reduction. Subsequently, the surface was finished with fine and superfine grain diamonds No 6879, CD720014 and CD72F012 (Busch & Co. KG, Engelskirchen, Germany) and was controlled under magnification glasses (2.7×).

### 4.3. Digital Data Acquisition

The prepared abutment teeth were dried and covered with a thin layer of scan spray (Xiondental, München, Germany) to obtain accurate scans (D 810; 3 shape, Copenhagen, Denmark). For the fabrication of the occlusal surface of the crown, casts of the appropriate antagonistic occlusal surface were made using K-Sillikon Eurosil, Max 2 (Henry Schein, New York, NY, USA). To obtain accurate digital scans, the antagonistic occlusal surface was covered with a thin layer of zirconium powder spray (Zfx SCAN; Xiondental, München, Germany). Digital data of the preparation and silicon cast were used for the crown design Version 14.82 (3shape, Copenhagen, Denmark). 

### 4.4. Full Crown Materials

The CAD/CAM crowns were fabricated from the following materials: resin nanoceramic Lava Ultimate (3M Espe, Seefeld, Germany); glass ceramic in a resin interpenetrating matrix Vita Enamic (Vita, Bad Säckingen, Germany); and lithium silicate reinforced ceramics enriched with ZrO_2_ Vita Suprinity (Vita, Bad Säckingen, Germany). The characteristics of the materials used are summarized in Table 7. 

### 4.5. Manufacturing and Adjustment of the Ceramic Crowns

STL-files of the crowns were sent to a dental milling center (DMC, Cologne, Germany), to Vita Zahnfabrik (VITA, Bad Säckingen, Germany) and to Amann Girrbach (Amann Girrbach, Koblach, Austria), where they were milled according to the standard manufacturer’s procedures; the crowns occlusal thickness was 1.5 mm. 

The fit of the corresponding full ceramic CAD/CAM crowns to the prepared abutment tooth was checked by one master dental technician before and after fitting regarding the marginal gap, rotation and the time required for the fitting. 

After fitting, the Vita Suprinity crowns were fired in a ceramic Dekema Autromat 3001 oven (DEKEMA, Freilassing, Germany) for 480 s at 840 °C. After fitting, all of the crowns were finished and polished by one operator according the manufacturer`s recommendations (Table 8), and the crowns were checked by a master dental technician.

### 4.6. Crown Cementation 

The prepared abutment teeth and the ceramic crowns were cleaned with a Steamer X3 (Amann Girrbach AG, Koblach, Austria) and were slightly air dried before cementation. No further conditioning of the abutment teeth or the ceramic crowns was applied. All of the crowns were cemented with self-adhesive dual-curing resin cement (RelyX Unicem 2 Automix; color A2, 3M Espe, Seefeld, Germany) according to the manufacturer’s instructions. The crowns were seated with finger pressure for ten seconds and were then axially loaded for 10 min with an adapter (Metal Dölz, Kraftsdorf/Harpersdorf, Germany) that ensured a defined axial load of 60 N (6 kg). Light curing was performed for two seconds per crown surface. After that, the excess cement was carefully removed with a foam pellet and scaler. Then, the pro crown surface was exposed to light curing and was exposed for 20 s again. The marginal fit of the crown was checked by visual inspection.

### 4.7. Chewing Simulation

First, the abutment tooth with the full CAD/CAM ceramic crowns and the natural antagonists were fixed in the correct occlusion with wax. Then, the abutment tooth with the crowns was attached using a Technovit 3040 (Heraeus Kulzer GmbH, Hanau, Germany) in the position intended for the chewing simulator pedestals and the natural antagonists were fixed in a special adapter using a Technovit 3040 (Heraeus Kulzer, Hanau, Germany). Between three and five days after crown cementation four pairs of teeth were fixed in a chewing simulator CS 4.8, SD (Mechatronics, Munich, Germany). The occlusal contacts were examined, and the contact points were monitored using occlusal paper and were recorded in a protocol. 

To simulate the oral cavity medium and to test the impact of the chewing force and the duration of chewing in the wet condition on the occlusal surface, the abutment tooth with CAD/CAM ceramic crowns and their antagonistic teeth were placed in the test chamber with 300 mL of artificial saliva with a pH of 7.0 (Dental center, Erfurt, Germany) at room temperature. The chewing simulation in a chewing simulator (Willytec CS 4.4, SD Mechatronics, Munich, Germany) simulated vertical jaw occlusion and laterotrusion. The site movement was 0.7 mm with a lateral speed of 20 mm/s, and the vertical movement was 5 mm with a vertical speed of 60 mm/s. The samples were loaded with 5 kg (50 N) for 1,200,000 cycles (Ivoclar wear method [5]).

After the chewing simulation, the occlusal surface of the ceramic crowns and the natural antagonists were cleaned with a Steamer X3 (Amann Girrbach AG, Koblach, Austria) and were slightly air dried.

### 4.8. Analysis of Parameters

#### 4.8.1. Surface Roughness Analysis 

The surfaces roughness measurement of the full ceramic CAD/CAM crowns occlusal surfaces was determined before and after the chewing simulation using the optical profilometry Infinite Focus System G3 (Alicona Imaging, Graz, Austria) with a vertical resolution of 450 nm and an Lc of 25. For the purpose occlusal surface scans of the full ceramic CAD/CAM crowns to generate STL files, three 50 µm^2^ areas were randomly selected, and three roughness measurement pro areas were performed. The roughness parameters were determined on these areas as Area-Scale Fractal Complexity (asfc) dimensionless values which is related to the fractal dimension of the surface texture [29]. The measuring procedure followed the ISO 11562 recommendations.

#### 4.8.2. Two-Dimensional Vertical Loss Analysis

To assess the vertical loss with regard to the cusp axis after chewing simulation, 3D scans of the occlusal surfaces of all of the crowns and the natural antagonist were made using a 3D dental scanner D810 (3Shape, Copenhagen, Denmark) with a resolution of 7 µm validated and documented according ISO 12836. The STL files were superimposed in the Infinite Focus System G3 (Alicona Imaging, Graz, Austria), and the vertical loss of the main abraded cusp was measured in µm. Adjustment of the superimposition of the STL files was automatically calculated by the program using the base of the specimens as normalized plane.

#### 4.8.3. Three-Dimensional Volume Loss Analysis

The volume loss for every crown and the natural antagonist was determined and analyzed using GOM Inspect V7.5 SR2 (GOM-Gesellschaft für Optische Messtechnik mbH, Braunschweig, Germany).

### 4.9. Evaluation of the Collected Data 

The measured data were analyzed using the Statistical Package for Social Sciences (SPSS) Version 22 (IBM, Armonk, NJ, USA). The inductive statistical analysis for normally distributed data were performed using Kruskal-Wallis, a Chi^2^ and McNemar-Bowker tests. Additionally, the parametrical analysis of variance was calculated. Significance was set at *p* < 0.05. The inductive statistical analyses for the non-parametric distributed data were carried out using the Mann-Whitney U test for independent variables and the Wilcoxon signed-rank test for related variables. Three tests were performed using the same data. A Bonferroni adjustment of the *p* value was applied and resulted in a final *p* value of 0.016. The descriptive data are presented as tables and boxplot graphs.

## 5. Conclusions

Using an appropriate ceramic CAD/CAM material opposite the natural antagonists promotes the effective durability of these two contact occlusal surfaces and the efficient function of the whole chewing system. For full ceramic CAD/CAM molar crowns, resin nanoceramic (Lava Ultimate) is the most natural antagonist-friendly material; however, it is susceptible to vertical and volume material loss. Glass ceramic in a resin interpenetrating matrix (Vita Enamic) is the most occlusion-stable material for full ceramic CAD/CAM molar crowns.

## Figures and Tables

**Table 1:** Descriptive analysis of the asfc values and results of the Mann-Whitney U test comparing the full ceramic computer-aided design/computer-aided manufacturing (CAD/CAM) crowns occlusal surface roughness in the tested material groups before and after the chewing simulation.

	Minimum	Median	Maximum	Minimum	Median	Maximum	
	before			after			*p* Value
Lava Ultimate	0.6	1.4	8.6	1.4	7.4	112	0.114
Vita Enamic	0.7	1.6	21.5	1.5	2.9	6.2	0.386
Vita Suprinity	0.9	5.2	24.2	0.4	5.4	25.3	0.721

**Table 2:** Results of the Mann-Whitney U test comparing the full ceramic CAD/CAM crowns occlusal surface roughness between the tested material groups before and after the chewing simulation.

	Vita Enamic Crown		Vita Suprinity Crown	
	before	after	before	after
Lava Ultimate crown	*p* = 1	*p* = 0.043	*p* = 0.143	*p* = 0.247
Vita Enamic crown			*p* = 0.165	*p* = 0.436

**Table 3:** Results of the Mann-Whitney U test comparing of the median value of the vertical loss of the full ceramic CAD/CAM crowns between the tested material groups.

	Vita Enamic Crown	Vita Suprinity Crown
Lava Ultimate crown	*p* < 0.001	*p* = 0.005
Vita Enamic crown	--	*p* = 0.002

**Table 4:** Results of the Mann-Whitney U test comparing of the median value of the vertical loss of the natural antagonists between the tested material groups.

	Vita Enamic Antagonist	Vita Suprinity Antagonist
Lava Ultimate antagonist	*p* = 0.019	*p* = 0.089
Vita Enamic antagonist	--	*p* = 0.853

**Table 5:** Results of the Mann-Whitney U test comparing of the median value of the total occlusal abrasive wear (volume loss) of the full ceramic CAD/CAM crowns between the tested material groups. Results of the comparison of the total abrasive wear between all of the materials.

	Vita Enamic Crown	Vita Suprinity Crown
Lava Ultimate crown	*p* < 0.001	*p* < 0.001
Vita Enamic crown		*p* < 0.023

**Table 6:** Results of the Mann-Whitney U test comparing of the median value of the total occlusal abrasive wear (volume loss) of the natural antagonists between the tested material groups. Results of the comparison of the total abrasive wear between all of the materials.

	Vita Enamic Antagonist	Vita Suprinity Antagonist
Lava Ultimate antagonist	*p* = 0.007	*p* = 0.579
Vita Enamic antagonist		*p* = 0.143

**Table 7:** Description of the full crown materials used in this study.

Material	Chemical Composition *	Indication	Processing	Manufacture
Lava Ultimate	80% by weight of nanoceramics (zirconia and silica nanoparticles) components that are embedded in a highly cross-linked polymeric matrix (20% by weight)	All definitive, adhesive single restorations: onlay, inlay, veneer	CAD/CAM Modifications and additions are possible: with a methacrylate-based light-curing restorative material for both intraoral and extraoral application	3M Espe, Seefeld, Germany
Vita Enamic	Hybrid ceramic with a dual network structure (glass ceramic in a resin interpenetrating matrix). The main feldspathic ceramic network (86% by weight) is reinforced with a polymer (UDMA, TEGDMA) (14% by weight)	Anterior and posterior crown; minimally invasive reconstructions: small defective non-prep-veneer tabletop	CAD/CAM will not be fired, polishing individualization by polymerization	Vita Zahnfabrik, Bad Säckingen, Germany
Vita Suprinity	Lithium silicate reinforced ceramic enriched (% by weight) with ZrO_2_ (8 to 12), SiO_2_ (56 to 64), Li_2_O (15 to 21), K_2_O (1 to 4), P_2_O_5_ (3 to 8), Al_2_O_3_ (1 to 4) and CeO_2_ (0 to 4)	Anterior and posterior crown, implant crown;veneer	CAD/CAM is reworked in the precrystallitezed state polishing, crystallization	Vita Zahnfabrik, Bad Säckingen, Germany

UDMA: urethane dimethacrylate; TEGDMA: triethylene glycol dimetharcylate; * a: according to the information provided by the manufacturers.

**Table 8:** Polishing protocol was performed according to the information provided by the manufacturers.

Lava Ultimate	Vita Enamic	Vita Suprinity
1. Cleaning of the restoration in an ultrasonic bath or steam jets. Drying with an air blower.	1. Cleaning the restoration in an ultrasonic bath or steam jets. Drying with an air blower.	1. Polishing was carried out with the diamond pink instruments at a speed from 7000 to 12,000 rotation/min.
2. Remove sprue with a coarse rubber wheel.	2. Ablating grinding pin with diamond grinding tool	2. Subsequently, the high-gloss polishing is carried out with the gray diamond tool at a reduced speed from 4000 to 8000 rotation/min.
3. Smooth the area of “Angus spins” with a medium-hard rubber wheel or a rubber tip.	3. Perform contouring, pre- and high-gloss polishing with the instruments of the Vita Enamic Polishing Set technical and clinical.	3. The heat development during pre-polishing and high-gloss polishing must be avoided. The reduced and uniform contact pressure is also very important.
4. Using of the polishing paste, incorporated in a “Robinson” brush.	4. Only the medium (M), fine (F) and very fine (SF) grained variants must be used. Grinding better to perform in wet conditions.	
5. Applying the polishing compound at low speed and with a handpiece on the restoration surface		
6. Polishing with the muslin rag wheel.		
7. Inner surface of the restoration sandblasting (aluminium dioxid grain with size of ≤50 microns with 2 bar pressure) until the inner surface lusterless appears.		
